# Identification of Novel 1,3,5-Triphenylbenzene Derivative Compounds as Inhibitors of Hen Lysozyme Amyloid Fibril Formation

**DOI:** 10.3390/ijms20225558

**Published:** 2019-11-07

**Authors:** Hassan Ramshini, Reza Tayebee, Alessandra Bigi, Francesco Bemporad, Cristina Cecchi, Fabrizio Chiti

**Affiliations:** 1 Biology Department, Payam Noor University, Tehran 19395-4697, Iran; ramshini@alumni.ut.ac.ir; 2 Department of Chemistry, School of Sciences, Hakim Sabzevari University, Sabzevar 96179-76487, Iran; rtayebee@hsu.ac.ir; 3 Section of Biochemical Sciences, Department of Biomedical, Experimental and Clinical Sciences, University of Florence, I-50134 Florence, Italy; alessandra.bigi@unifi.it (A.B.); francesco.bemporad@unifi.it (F.B.); cristina.cecchi@unifi.it (C.C.)

**Keywords:** protein misfolding, HEWL amyloid aggregation, drug discovery, small aromatic compounds, amyloid inhibitors, triphenylbenzene

## Abstract

Deposition of soluble proteins as insoluble amyloid fibrils is associated with a number of pathological states. There is a growing interest in the identification of small molecules that can prevent proteins from undergoing amyloid fibril formation. In the present study, a series of small aromatic compounds with different substitutions of 1,3,5-triphenylbenzene have been synthesized and their possible effects on amyloid fibril formation by hen egg white lysozyme (HEWL), a model protein for amyloid formation, and of their resulting toxicity were examined. The inhibitory effect of the compounds against HEWL amyloid formation was analyzed using thioflavin T and Congo red binding assays, atomic force microscopy, Fourier-transform infrared spectroscopy, and cytotoxicity assays, such as the 3-(4,5-Dimethylthiazol)-2,5-Diphenyltetrazolium Bromide (MTT) reduction assay and caspase-3 activity measurements. We found that all compounds in our screen were efficient inhibitors of HEWL fibril formation and their associated toxicity. We showed that electron-withdrawing substituents such as –F and –NO_2_ potentiated the inhibitory potential of 1,3,5-triphenylbenzene, whereas electron-donating groups such as –OH, –OCH_3_, and –CH_3_ lowered it. These results may ultimately find applications in the development of potential inhibitors against amyloid fibril formation and its biologically adverse effects.

## 1. Introduction

The conversion of peptides or proteins from a soluble state into insoluble, β-sheet rich, fibrillar aggregates, generally called amyloid fibrils, is a key event in a large family of human pathologies, including neurodegenerative and non-neuropathic systemic amyloidosis [1,2]. Among these, Alzheimer’s disease (AD) is the most known and diffuse and it has an increasing incidence in the worldwide population. Similarly, Parkinson’s disease (PD), as well as Huntington’s, prion diseases, type II diabetes, and systemic amyloidosis share the common hallmark of amyloid fibrillar aggregates [1,2,3]. Although the proteins that comprise these deposits do not share any sequence or structural homology, amyloid fibrils formed by different proteins share similar structural and physicochemical properties and, possibly, a common pathogenic mechanism [4,5]. These diseases involve self-assembly of soluble proteins into large insoluble fibrils through nucleation-dependent assembly, often via the formation of oligomeric structures that possess toxic properties [1,6,7]. Therefore, preventing oligomer and fibril formation appears an attractive approach to tackling the progress of such diseases.

Fibril formation is a polymerization process which can be simply described by a sigmoid curve with three phases, originally suggested to be a three-stage process consisting of nucleation, fibril elongation, and the final plateau [8], although the most recent progress has elucidated that each of the three phases involve a combination of the various microscopic steps [9,10]. Despite intensive efforts to treat amyloid-associated diseases, there is no fully effective and definitive treatment of such diseases [1,2,11,12]. Several strategies have been undertaken in the attempt to interfere with the formation and deposition of amyloid fibrils and their pathogenic role, including inhibition of the formation of the amyloidogenic form of proteins by stabilization of the native conformation, reduction in the production of the amyloidogenic sequence from a more complex protein, inhibition of protein self-assembly in oligomers and fibrils, and enhancement in the clearance of toxic aggregates [13,14,15,16,17].

The propensity of proteins and peptides to form amyloid fibrillar structures is correlated to their physicochemical properties such as net charge, secondary structure tendency, hydrophobicity, and aromatic interactions [18,19]. Regions of the sequence of proteins promoting amyloid fibril formation are often enriched with aromatic residues [20,21,22]. There are different hypotheses on the precise role of the aromatic residues in the amyloidogenic process. One of them suggests that aromatic amino acid residues promote peptide self-assembly for their hydrophobic nature, high β-sheet propensity and null charge, along with their planar geometry that are the most relevant factors in self-assembly [23,24,25,26,27]. According to this hypothesis, the aromatic nature of these residues does not contribute significantly to their amyloidogenicity. Other various studies suggest that aromatic amino acids contribute to peptide self-assembly through π-stacking interactions [22,28,29]. Other groups also believed that both hydrophobicity and aromaticity play a role in the self-assembly [30]. Regardless of the precise mechanism by which aromatic residues drive amyloid fibril formation, the parallel in-register alignment of β-strands in amyloid fibrils contributes to create a stacking of aromatic rings along the fibril axis. Thus, aromatic compounds may competitively interact with aromatic residues in amyloidogenic proteins, preventing their stacking, and block the self-assembly process [31]. It has been found that the effect of aromatic compounds on amyloid aggregation strongly depends on their structure and functional groups [32,33]. Multiple studies have reported that the number of phenyl rings and electron-withdrawing or -donating groups on the molecules, as well as their positions, are important characteristics that determine the effectiveness of the aromatic compounds in their inhibition of Aβ aggregation [34,35,36]. Following this concept, six aromatic molecules belonging to the category of 1,3,5-triphenylbenzene and having different electron-donor or -withdrawing substituents, have been synthesized and their possible effects on amyloid fibril formation by a sample protein have been tested. As a sample protein we have chosen hen egg white lysozyme (HEWL), as it is known to form amyloid fibrils under appropriate conditions [37,38,39,40] and because the properties of the resulting fibrils and the of the overall amyloid formation process have been investigated to a significant extent [41,42]. The effects of the compounds on amyloid fibril formation by HEWL and on the biological properties of the resulting aggregates have been investigated through the use of various techniques, including Congo red (CR) and thioflavin T (ThT) assays, Fourier-transform infrared (FTIR) spectroscopy, atomic force microscopy (AFM), the MTT reduction assay, and caspase activity measurements. Our results showed the importance of the substituents on the efficiency of aromatic compounds, with compounds containing electron-withdrawing substituents having greater efficacy in HEWL amyloid inhibition, thus representing promising compounds for developing inhibitors of amyloid fibril formation.

## 2. Results

### 2.1. Chemistry of the Compounds

**Scheme 1:**
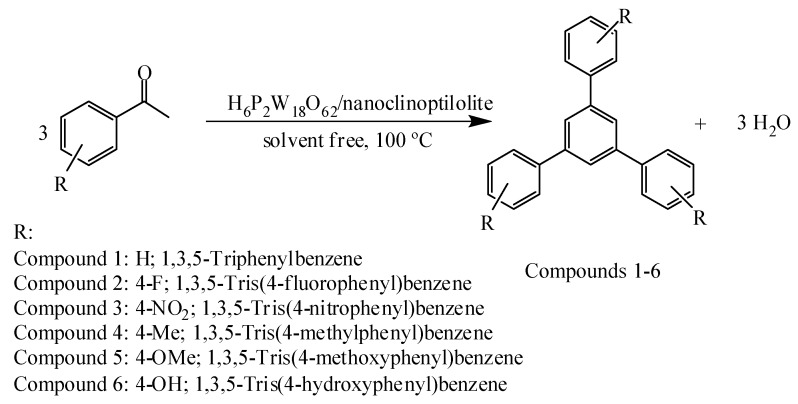
General formulation for the cyclotrimerization of acetophenones.

Scheme 1 shows a general formula for the preparation of some para-substituted 1,3,5-triphenylbenzenes (compounds **1**–**6**). These pharmaceutical important compounds are made by the condensation of aryl methyl ketones catalyzed by HPA/NCP under solvent-free conditions. Nanoclinoptilolite (NCP) refers to a natural zeolite composed of a microporous arrangement of silica and alumina and also HPA (H_6_P_2_W_18_O_62_) refers to the Wells–Dawson heteropolyacid. The heteropolyacid catalyst activates the carbonyl group toward the electrophilic substitution. Using this procedure, six compounds belonging to the category of para-substituted 1,3,5-triarylbenzenes have been synthesized and purified as previously described [43,44]. These are 1,3,5-triphenylbenzene (Compound **1**); 1,3,5-tris(4-fluorophenyl)benzene (Compound **2**); 1,3,5-tris(4-nitrophenyl)benzene (Compound **3**); 1,3,5-tris(4-methylphenyl)benzene (Compound **4**); 1,3,5-tris(4-methoxyphenyl)benzene (Compound **5**); and 1,3,5-tris(4-hydroxyphenyl)benzene (Compound **6**).

The names and chemical formulas of the six compounds are reported in Scheme 1. Their NMR spectra are reported in the supplementary information, again along with their chemical formulas (Figures S1–S6).

### 2.2. Effect of 1,3,5-Triarylbenzenes and Their Derivatives on Heat-Induced Fibrillation of HEWL Monitored with ThT Fluorescence

**Figure 1:**
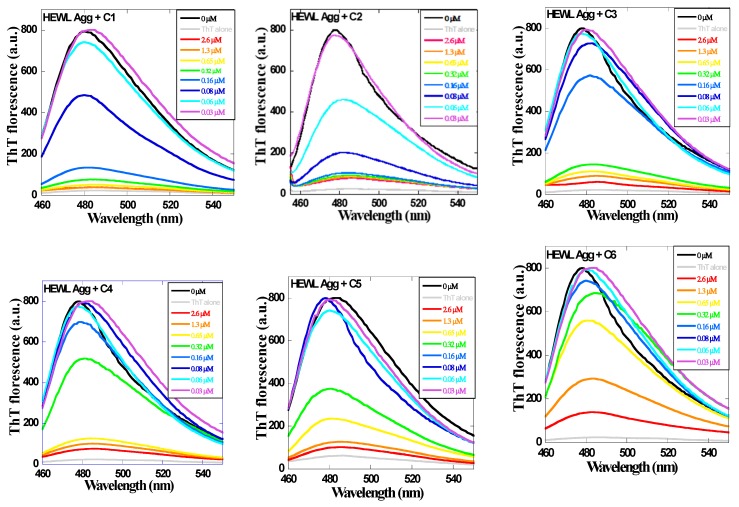
Dose-dependent inhibition of 0.03–2.6 µM compounds **1**–**6** on hen egg white lysozyme (HEWL) amyloid fibril formation at 2 mg/mL (140 µM), in 50 mM glycine buffer, pH 2.5, and 57 °C, under stirring (250 rpm), for 48 h, monitored by changes in thioflavin T (ThT) florescence intensity. The spectra refer to ThT alone (gray), HEWL pre-incubated with 0 (black), 0.03 (violet), 0.06 (indigo), 0.08 (dark blue), 0.16 (blue), 0.32 (green), 0.65 (yellow), 1.3 (orange), and 2.6 (red) µM of compounds **1**–**6**.

**Figure 2:**
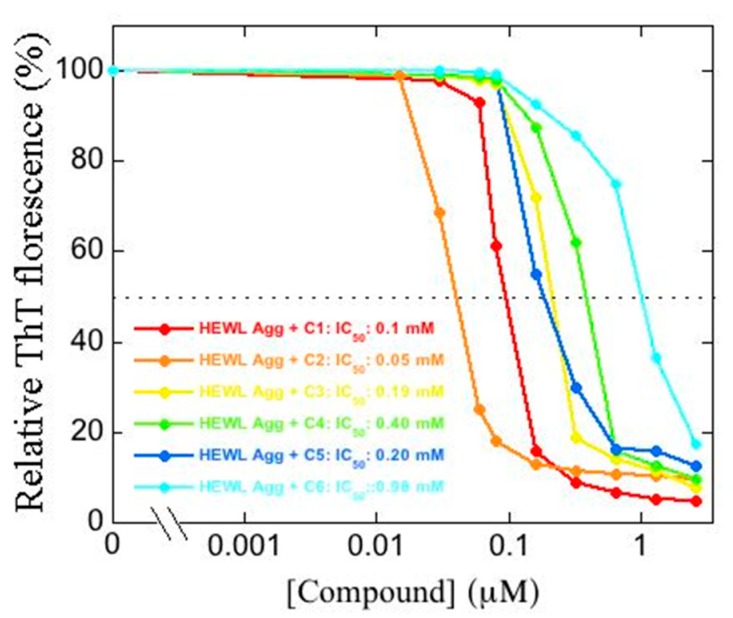
Concentration-dependent prevention of HEWL amyloid fibril formation by compounds **1**–**6** incubated as described in Figure 1 at different compound concentrations, ranging from 0.03 to 2.6 µM. The traces refer to compound **1** (red), compound **2** (orange), compound **3** (yellow), compound **4** (green), compound **5** (blue), and compound **6** (indigo). The dotted line indicates a level of 50%.

The inhibition potential of the six compounds was determined using the ThT fluorescence assay to measure the amount of amyloid aggregates after the addition of various concentrations of each compound ranging from 0.03 to 2.6 µM. Aggregation of HEWL was induced by incubating the protein for 48 h at a concentration of 2 mg/mL (140 µM) in 50 mM glycine buffer, pH 2.5, and 57 °C, while stirred gently by magnetic bars (rpm 250), in the absence or presence of 0.03–2.6 µM compounds **1**–**6**. All compounds clearly showed a pattern of dose-dependent inhibition, as suggested by the observed decrease of ThT fluorescence intensity (Figure 1). The IC_50_ values of the compounds, corresponding to the concentration at which the inhibitory effect is half of the maximum effect, were determined (Figure 2). The relative order of efficacy of the compounds in terms of IC_50_ value is compound **2** > compound **1** > compound **3** ~ compound **5** > compound **4** > compound **6** and, thus, compound **2** is the best inhibitor and compound **6** has minimum effect in this process. As shown in Figure 1 and Figure 2, 0.32 µM of all compounds significantly inhibited the aggregation of HEWL, thus this concentration was used for the subsequent experiments.

A control experiment was also designed to rule out the possibility that the low ThT fluorescence intensities measured in the presence of these compounds might result from the quenching of ThT fluorescence by the compounds themselves. These compounds were separately added at final concentrations of 2.6 μM to pre-formed HEWL aggregates and the ThT fluorescence assay was performed immediately before and after compound addition. The ThT fluorescence values were found to be similar in each case, before and after compound addition, indicating the lack of any significant quenching effect (Figure S7).

### 2.3. Effect of 1,3,5-Triarylbenzenes and Their Derivatives on Heat-Induced Fibrillation of HEWL Monitored with CR Assay

**Figure 3:**
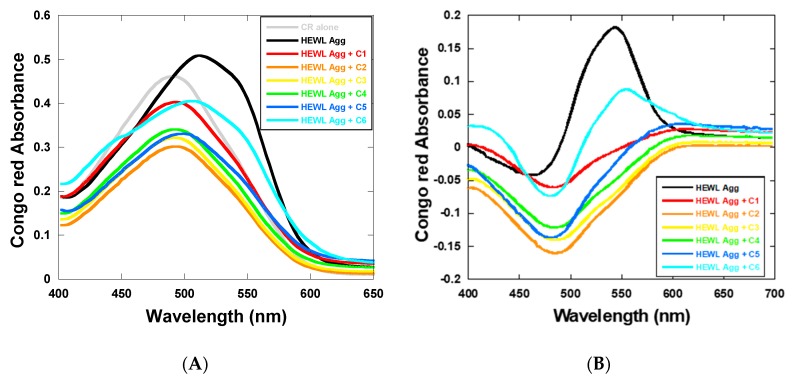
Effects of compounds **1**–**6**, at a concentration of 0.32 µM, on HEWL aggregation at 2 mg/mL, 50 mM glycine buffer, pH 2.5, 57 °C, under stirring (250 rpm), monitored by changes in CR absorbance after 48 h. (**A**) The spectra refer to CR alone (gray), HEWL pre-incubated under aggregating conditions without compounds (black), with compound **1** (red), compound **2** (orange), compound **3** (yellow), compound **4** (green), compound **5** (blue), and compound **6** (indigo). (**B**) Difference spectra obtained by subtracting the spectrum of CR alone from the spectra of HEWL aggregates with CR reported in (**A**). Color code as in (**A**).

Aliquots of the same samples used for ThT experiments were also used to carry out the CR assays. The CR absorbance measured in the absence of HEWL and any of the compounds was found to be low, with a peak at 490 nm (Figure 3A). The CR absorbance measured in the presence of HEWL pre-incubated for 48 h under aggregating conditions in the absence of any compound exhibited a weak increase accompanied by a red shift of the peak to 520–540 nm (Figure 3A). The higher CR absorbance obtained in the presence of HEWL is likely to originate from the presence of protein aggregates that scatter light, whereas the red shift of the peak from 490 to 520–540 nm indicates the presence of β-sheet containing aggregates. The CR absorbance measured in the presence of HEWL pre-incubated for 48 h under aggregating conditions with 0.32 µM compounds **1**, **2**, **3**, **4**, and **5** were found to be lower than that measured in the absence of the compounds (Figure 3A). This indicates that these compounds were able to inhibit HEWL amyloid fibril formation. The CR absorbance measured in the presence of HEWL and 0.32 µM compound **6** was found to be markedly higher, with a higher red shift, indicating that this compound was not effective in inhibiting HEWL amyloid formation. These results were confirmed by analyzing the difference spectra, obtained by subtracting in each case the spectrum of CR alone from those obtained with HEWL aggregates and CR (Figure 3B). The absorbance peak at 540 nm, obtained without compounds **1**–**6** and readily assigned to CR bound to the aggregates, was found to be suppressed in the presence of the various compounds **1**–**5**, with only a modest decrease in the presence of compound **6**.

In this study, we used dimethyl sulfoxide (DMSO) for dissolving our compounds, and, thus, a control experiment was carried out to determine whether DMSO itself had an effect on the HEWL aggregation. However, 2% (*v*/*v*) DMSO used here has a minimal effect on the aggregation of HEWL (Figure S8). Therefore, all compounds were dissolved in DMSO prior to use.

The results observed by the ThT and CR assay depicted in Figure 1, Figure 2 and Figure 3 could be due to an ability of the six compounds to inhibit HEWL aggregation. Alternatively, they might just inhibit amyloid fibril formation without preventing the formation of protein aggregates with a non-amyloid structure. To assess either possibility, we centrifuged the various HEWL samples and measured the protein concentration of the resuspended pellets, showing that pellets obtained from samples preincubated with compounds **1**–**6** had a higher protein content than those incubated without compounds. This indicated that the aromatic molecules did not inhibit aggregation of HEWL generally, but more specifically its process of amyloid fibril formation.

### 2.4. Effect of 1,3,5-Triarylbenzenes and Their Derivatives on Heat-Induced Fibrillation of HEWL Monitored with FTIR Spectroscopy

**Figure 4:**
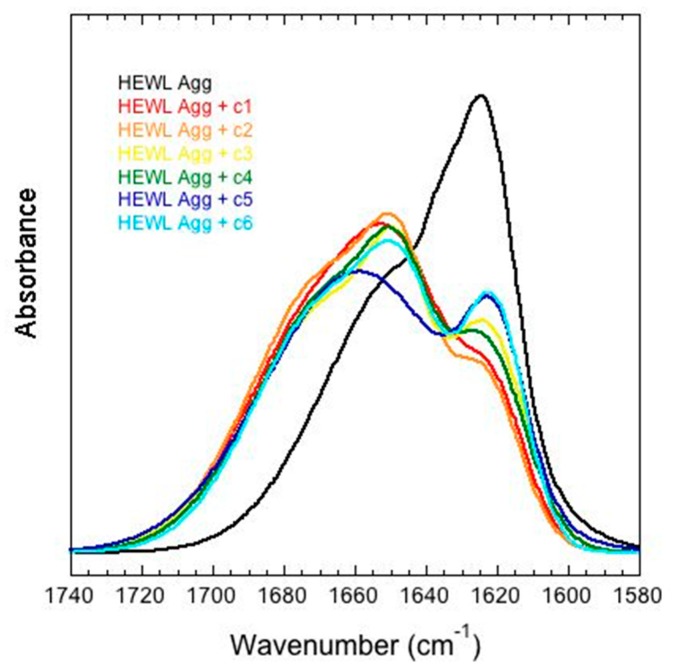
Inhibitory effects of the compounds **1**–**6** on the formation of β-sheet secondary structure of HEWL monitored by FTIR. HEWL amyloid fibril formation was pre-incubated at 2 mg/mL (140 µM), in 50 mM glycine buffer, pH 2.5, and 57 °C, under stirring (250 rpm), for 48 h in the presence of 0.32 µM compounds **1**–**6**, centrifuged at 13,000× *g* for 10 min and resuspended in D_2_O to a final HEWL concentration of 27.8 mg/mL. Blank-subtracted, baseline-corrected, and normalized FTIR spectra (amide I region) of HEWL aggregates without (black) or with compound **1** (red), compound **2** (orange), compound **3** (yellow), compound **4** (green), compound **5** (blue), and compound **6** (indigo).

New samples of HEWL aggregates with or without compounds **1**–**6** at a concentration of 0.32 µM were prepared, under the same conditions as those used for ThT and CR experiments, centrifuged after 48 h to collect the aggregates, resuspended at high concentration in D_2_O and analyzed with FTIR spectroscopy, to determine the effects of the six compounds on the secondary structure of the HEWL aggregates. The HEWL aggregates without compounds showed a clear maximum at 1624 ± 1 cm^−1^ in the amide I region of the FTIR spectrum, which can readily be assigned to intermolecular β-sheet structure (Figure 4). Another peak is present at ca. 1650 cm^−1^ attributable to either unordered or α-helical secondary structure. All the HEWL aggregates formed in the presence of the compounds showed a lower absorbance peak at 1623–1625 cm^−1^ and a higher absorption at 1648–1652 cm^−1^, indicating loss of β-sheet structure and gain of other types of secondary structure in the aggregates (Figure 4). In particular, compound **2** led to the most remarkable change, followed by compound **1**, compound **4**, compound **3**, compound **5**, and compound **6**, a ranking similar to that observed with ThT fluorescence.

### 2.5. Effect of the Compounds on the Kinetic of HEWL Aggregation

**Figure 5:**
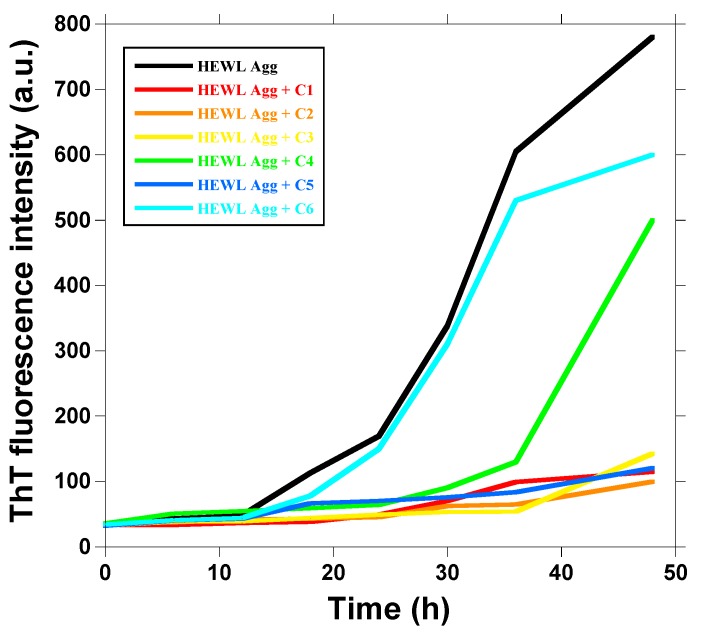
Kinetics of HEWL aggregation at 2 mg/mL (140 µM), in 50 mM glycine buffer, pH 2.5, 57 °C, under stirring (250 rpm), in the presence of 0.32 µM of compounds **1**–**6** followed by monitoring ThT fluorescence intensity. The kinetic traces refer to HEWL without compounds (black), HEWL with compound **1** (red), HEWL with compound **2** (orange), HEWL with compound **3** (yellow), HEWL with compound **4** (green), HEWL with compound **5** (blue), and HEWL with compound **6** (indigo).

The time course of HEWL amyloid fibril formation in the absence of the compounds monitored by measuring the ThT fluorescence emission intensity over a period of 48 h was nucleation dependent, with a typical sigmoidal profile, including lag, exponential, and equilibrium phases [8]. This kinetic trace was meant to be qualitative rather than quantitative and, for this reason, data were acquired only every 6 h until 48 h. Inhibition of amyloid aggregation by the various inhibitors is generally quantified by assessing changes of the lag time, exponential phase, and aggregation extent at equilibrium plateau. The lag times measured in the presence of compounds **1**–**5**, thus except compound **6**, was relatively long (Figure 5). In addition, in presence of all compounds, again except compound **6**, the exponential phase changed but with different magnitudes (Figure 5).

### 2.6. Effect of the Compounds on HEWL Aggregation Morphology

**Figure 6:**
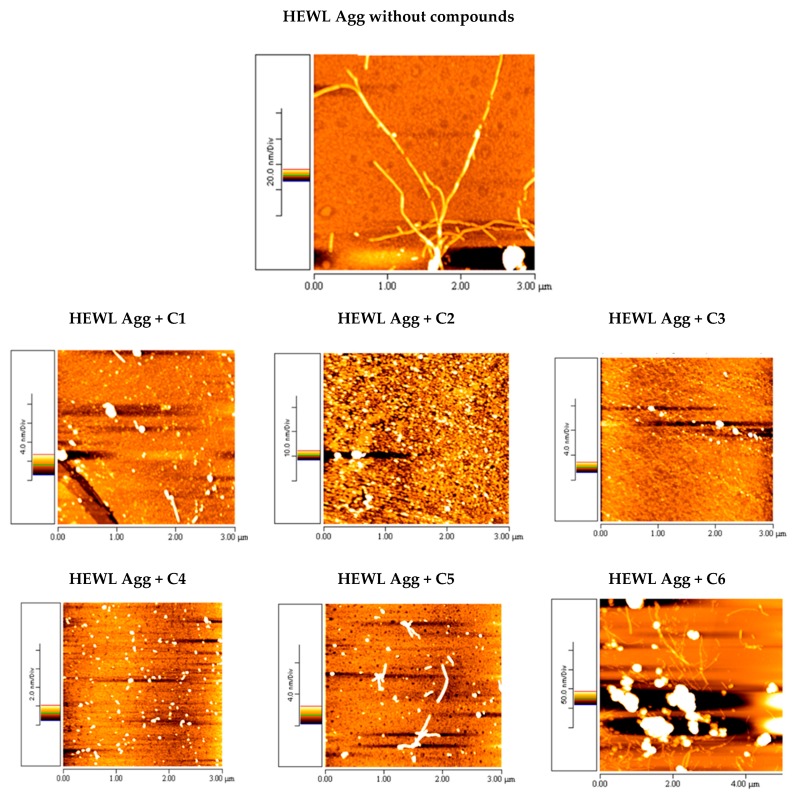
Atomic force microscopy (AFM) images of HEWL aggregates formed in absence or presence of compounds **1**–**6**. HEWL was pre-incubated under conditions promoting amyloid fibril formation that is 48 h at a concentration of 140 µM, in 50 mM glycine buffer, pH 2.5, 57 °C, under stirring (250 rpm), in the absence or presence of 0.32 µM compounds **1**–**6**.

In order to define further the nature of the aggregated species, an analysis of their morphologies was carried out using AFM. HEWL was incubated under the same aggregation conditions used for the ThT, CR, and FTIR experiments, i.e., for 48 h at a concentration of 2 mg/mL (140 µM) in 50 mM glycine buffer, pH 2.5, and 57 °C, under stirring (250 rpm), in the absence or presence of 0.32 µM of compounds **1**–**6** and the resulting samples were examined by AFM (Figure 6). HEWL aggregates in the absence of compounds showed typical long, unbranched fibrils. On the other hand, when HEWL was incubated in presence of compounds **1**–**4**, very few fibrils were observed after 48 h. Rather, oligomeric and/or amorphous species were predominant. HEWL pre-incubated in presence of compound **5** showed a few short fibrillar assemblies along with oligomeric species and, in the presence of compound **6**, a mixture of unbranched filamentous assemblies appeared. These results again showed that compound **6** cannot inhibit the formation of HEWL aggregates in agreement with the ThT and CR results.

### 2.7. Effect of the Compounds on HEWL Amyloid Induced Cytotoxicity

**Figure 7:**
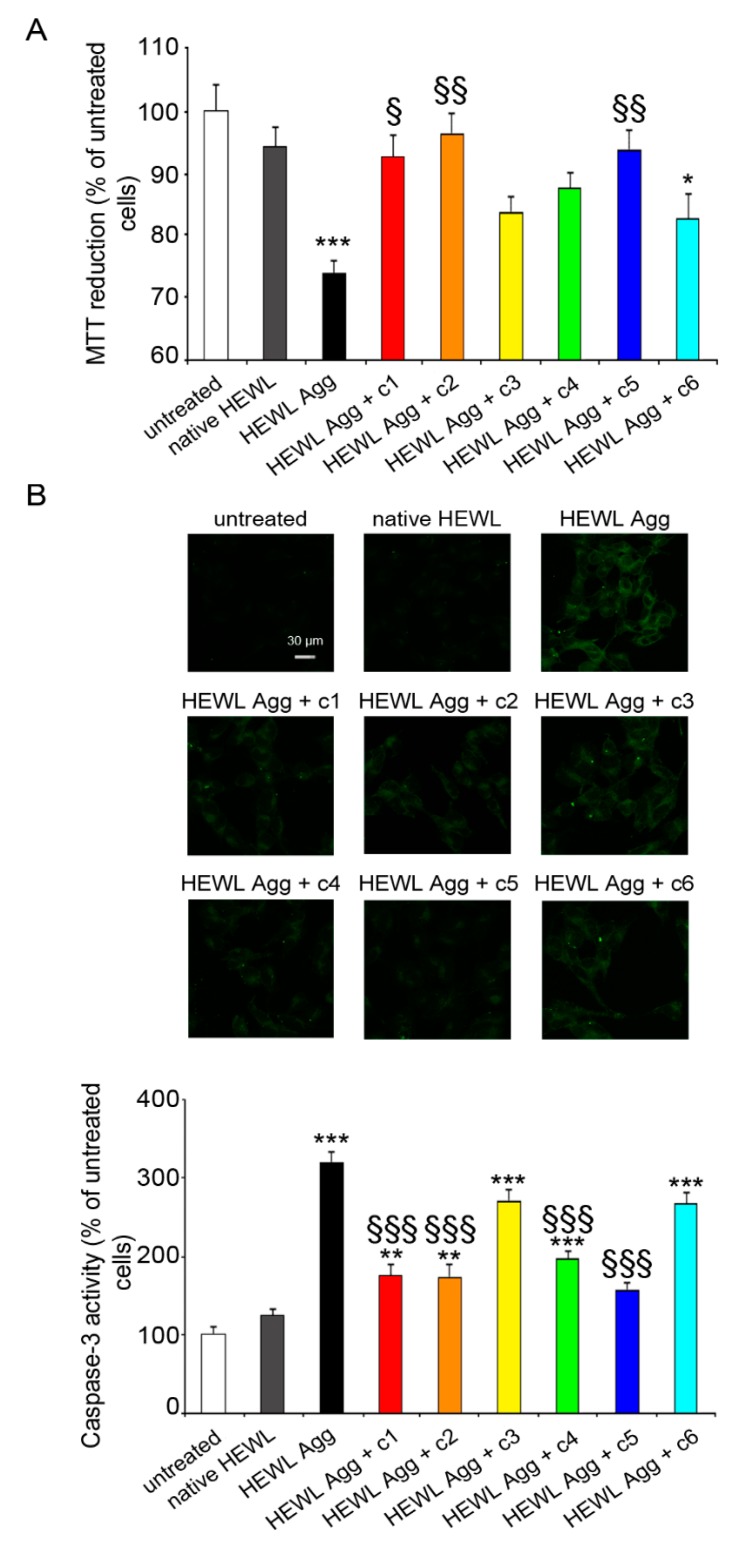
Impairment of cell viability after exposure to HEWL aggregates. (**A**) MTT reduction in SH-SY5Y cells treated for 24 h with 2 µM (monomer equivalents) HEWL aggregates formed in the absence or presence of the indicated compounds. (**B**) Caspase-3 activity in SH-SY5Y cells treated for 24 h with 2 µM HEWL aggregates formed in the absence or presence of the indicated compounds. The green fluorescence arises from intracellular caspase-3 activity. The results of a semi-quantitative analysis of the caspase-3-derived fluorescence data are expressed as the percentage of the value for untreated cells. In both panels, experimental errors are standard error of mean (S.E.M). Samples were analyzed by one-way ANOVA followed by Bonferroni’s multiple comparison test relative to untreated cells (* *p* < 0.05, ** *p* < 0.01, *** *p* < 0.001), or to cells treated with HEWL aggregates in the absence of compounds (§ *p* < 0.05, §§ *p* < 0.01, §§§ *p* < 0.001).

We then assessed the cytotoxicity of HEWL aggregates, formed in the absence or presence of compounds **1**–**6**, according to the aggregation protocol described for the ThT experiments. HEWL aggregates were collected by centrifugation, dried under N_2_, dissolved in cell culture medium, and added to SH-SY5Y human neuroblastoma cells at a concentration of 2 µM (monomer equivalents). The ability of the resulting aggregates to cause cellular dysfunction was assessed by performing the MTT reduction inhibition assay, a widely used indicator of mitochondrial dysfunction [45]. The ability of SH-SY5Y cells to reduce MTT was significantly decreased to 73.9 ± 2.1% (relative to untreated cells) after a 24 h exposure to HEWL aggregates, whereas it was unaffected by native HEWL treatment (Figure 7A). The ability of cells to reduce MTT was significantly increased when HEWL aggregates were formed in the presence of compound **1** (to 92.7 ± 3.2%), 2 (to 96.2 ± 3.2%) and 5 (to 93.9 ± 2.9%). By contrast, incubation with compounds **3**, **4**, and **6** resulted in a partial rescue of the cell viability. Very similar results were obtained by measuring the activity of caspase-3, a well-recognized apoptotic marker [46]. A significant activation of caspase-3 (320 ± 12%, as compared to untreated cells) was observed following treatment for 24 h of SH-SY5Y cells with HEWL aggregates at 2 µM (Figure 7B). Moreover, the presence of compounds **1**, **2**, **4**, and **5** during HEWL aggregation significantly prevented the apoptotic response induced by aggregates (Figure 7B). Furthermore, in this case, compounds **3** and **6** generated only a slight protective effect on apoptotic pathway activation.

## 3. Discussion

Design and synthesis of novel small-compound inhibitors of the process of amyloid fibril formation is one of the leading therapeutic approaches for the treatment of amyloid diseases [47,48,49,50]. The studies on different amyloidogenic proteins showed that small aromatic compounds are able to inhibit fibril formation, possibly due to their ability to interfere with the β-sheet architecture of the fibrils and/or aromatic stacking in fibril stabilization [22,30,51,52]. In fact, the exact contribution of aromaticity to amyloid formation is controversial and remains under debate [53].

Various findings demonstrate that the functional groups appended to the aromatic rings [54,55], geometry and steric profile of the functional groups on the aromatic rings [53], and also number of rings [36] can dramatically affect amyloid formation. We have recently reported that bis(indolyl)phenylmethane compounds inhibited the formation of amyloid fibrils and associated toxicity in a concentration-dependent manner [33]. We also showed that all tested compounds were able to inhibit HEWL amyloid fibril formation but with different efficiencies, which showed the importance of slight variations of the functional groups within the same scaffold as a small molecule [33].

In this work, a series of novel 1,3,5-triarylbenzenes derivatives having four aromatic rings and different electron-donor or -acceptor substitutions were synthesized and evaluated for their potential to prevent HEWL amyloid aggregation. These compounds, similar to bis(indolyl)phenylmethanes studied previously, were shown to inhibit HEWL amyloid fibril formation under conditions in which the protein is initially partially unfolded, but with different magnitude. In particular, considering the various techniques used here, including the ThT and CR assays, AFM, and FTIR, compounds **1**–**3** were found to be clearly more effective as inhibitors in comparison to compounds **4**–**6**, particularly the latter that did not show any detectable effect (Figure 1, Figure 2, Figure 3, Figure 4 and Figure 5). These findings showed that electron-donating substituents have less efficacy in preventing HEWL amyloid formation, whereas electron-withdrawing groups have much more influence in amyloid inhibition. The IC_50_ values of the compounds were between 0.05 and 0.98 µM, which are two orders of magnitude lower than the IC_50_ value measured by a representative bis(indolyl)phenylmethane, i.e., 12.3 ± 1 µM [33].

The amorphous and unstructured HEWL aggregates formed in the presence of all compounds were less toxic than the HEWL fibrils formed in their absence, as shown by MTT cell viability and caspase activity assays (Figure 7). Moreover, our results showed that HEWL aggregates formed in the presence of 1,3,5-triarylbenzenes derivatives, similar to results obtained with bis(indolyl)phenylmethane [33] and curcumin [56] derivatives, were non-toxic or retain a weak toxicity, allowing all these compounds to be classified as class I molecules, based on the classification proposed by Ladiwala et al., i.e., molecules that remodel soluble oligomers into species (often large off-pathway aggregates) that are non-toxic [57].

It has been proposed that π–π stacking of aromatic rings can be enhanced when two rings are electron poor and rich, respectively [36]. Our work and results presented by other scientists [36] are in agreement with this theory, as the addition of a strong electron-withdrawing group, such as –NO_2_, –F, or –CN [36] on the aromatic inhibitor increases the inhibitory effect, possibly due to the formation of a strong electron donor–electron acceptor complex between the inhibitory compounds and the aromatic residues of HEWL. In particular, the –F group was found to be the most effective, probably due to its high electronegativity, followed by the –NO_2_ group. On the other hand, when an electron-donating group, such as a –OH, –CH_3_, or –OCH_3_, was added, amyloid inhibition is decreased due to a repulsion between the electron-rich aromatic ring of the inhibitor and the electron-rich aromatic residues of HEWL, which does not allow the establishment of a stable complex between the inhibitor and the protein undergoing aggregation. In this context, the –OH group is the least effective in inhibiting HEWL aggregation.

Inhibitory effects of bis(indolyl)phenylmethanes may be discussed from different points of view, including different factors, such as the importance of inter- and intra-molecular hydrogen bonds, electronic effects and steric hindrances, the intrinsic steric congestion originating from the structural framework of bis-indolylarylmethanes, all affected by the substituents. In the case of 1,3,5-triarylbenzenes, a fully planar formula presents three aryl substituents in the same plane as the central benzene ring. In this case, only the aryl substituents may exert a little steric congestion around the aryl rings. Therefore, the net structure should have a planar identity and we can assume an effective diffusion of these types of compounds into amyloid fibrils. This can explain both the most effective inhibitory potential of 1,3,5-triarylbenzenes relative to bis-indolylarylmethanes and the critical role played by the electron releasing/donating substituents on the aryl rings in determining such an inhibition.

## 4. Materials and Methods

### 4.1. Materials

Hen egg white lysozyme (HEWL), thioflavin T (ThT), and Congo red (CR) were purchased from Sigma-Aldrich (St. Louis, MO, USA). Other reagents were purchased from Merck (Darmstadt, Germany), unless stated otherwise. All synthesized molecules were compared, in terms of their spectral and physical data, with those reported previously [58].

### 4.2. General Chemistry

All reagents and starting materials were commercially available and were used as received. Natural clinoptilolite-rich tuffs were obtained from the Sabzevar region in the north-east of Iran. Fourier-transform infrared (FT-IR) spectra were recorded on a 8700 Shimadzu Fourier-transform spectrophotometer (Shimadzu, Tokyo, Japan) in the region of 400–4000 cm^−1^ using KBr pellets. ^1^H and ^13^C NMR spectra were recorded on a Bruker AVANCE 300 MHz instrument (Bruker, MA, USA) using TMS as internal reference. All products were identified by comparison of their spectral and physical data with those previously reported. Wells–Dawson diphosphooctadecatungstic acid H_6_P_2_W_18_O_62_·24H_2_O was prepared according to the literature method [59].

#### General Procedure for the Conversion of Acetophenone into 1,3,5-Triphenylbenzene

In a typical reaction, acetophenone (1 mmoL) and the surface-modified HPA/NCP (15 mg) were added to a small test tube and the reaction mixture was stirred for 2.5 h at 100 °C. After completion of the reaction, as indicated by thin layer chromatography (TLC), the reaction mass was cooled to 25 °C; then, hot ethanol was added to the reaction mixture and the mixture was stirred for 5 min. The insoluble catalyst was isolated via simple filtration. The filtrate containing water as the only byproduct of the cyclotrimerization was concentrated under reduced pressure, and finally the obtained crude product was purified through re-crystallization in EtOH:H_2_O (3:1) as confirmed by an intense single spot in TLC. The pure products were specified based on the spectral data and determination of their melting points.

##### 1,3,5-Triphenylbenzene (Compound **1**)

Light yellow solid; m. p. 171–173 °C. IR (KBr): v_max_ = 3053, 1651, 1494, 1431, 1125, 758, 700 cm^−1^. ^1^H NMR (DMSO-d_6_, 500 MHz): δ 7.88 (s, 3H), 7.87–7.86 (m, 6H), 7.52–7.49 (t, J = 7.5 Hz, 6H), 7.43–7.40 (t, J = 7.5 Hz, 3H).^13^C NMR (DMSO-d_6_, 125 MHz): δ 141.6, 140.1, 128.9, 127.7, 127.2, 124.4.


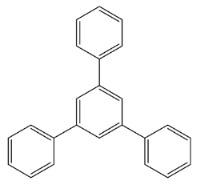


##### 1,3,5-Tris(4-Fluorophenyl)Benzene (Compound **2**)

White solid; m. p. 238–240 °C. IR (KBr): v_max_ = 3059, 1604, 1508, 1449, 1224, 1159, 825, 771 cm^−1^. ^1^H NMR (CDCl_3_, 500 MHz): δ 7.67 (s, 3H), 7.61 (d, J = 8.5 Hz, 6H), 7.59 (d, J = 8.5 Hz, 6H). ^13^C NMR (CDCl_3_, 125 MHz): 161.7, 137.6, 132.4, 129.0 (d, J = 7.5 Hz), 125.3, 115.9 (d, J = 22.5 Hz).


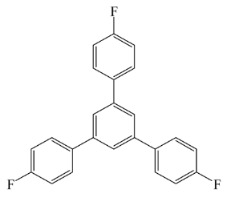


##### 1,3,5-Tris(4-Nitrophenyl)Benzene (Compound **3**)

White solid; m. p. 151–152 °C. IR (KBr): v_max_ = 3043, 1595, 1489, 1379, 1085, 1007, 849 cm^−1^. ^1^H NMR (CDCl_3_): δ = 7.56 (d, J 8.1 Hz, 6H), 7.62 (d, J 8.2 Hz, 6H), 7.70 (s, 3H). ^13^C NMR (125 MHz, CDCl_3_): δ 122.3, 125.0, 128.9, 132.1, 139.7, 141.6.


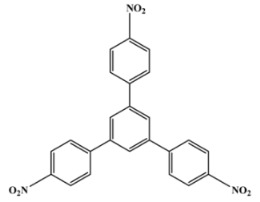


##### 1,3,5-Tris(4-Methylphenyl)Benzene (Compound **4**)

White solid; m. p. 178–179 °C. IR (KBr): v_max_ = 3017, 2922, 1612, 1511, 1491, 1109, 812 cm^−1^. ^1^H NMR (CDCl_3_, 500 MHz): δ 7.74 (s, 3H), 7.62 (d, J = 8.0 Hz, 6H), 7.30 (d, J = 7.5 Hz, 6H), 2.44 (s, 9H). ^13^C NMR (CDCl_3_, 125 MHz): 142.2, 138.3, 137.4, 129.4, 127.3, 124.7, 21.1.


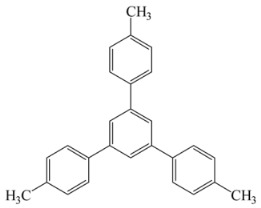


##### 1,3,5-Tris(4-Methoxyphenyl)Benzene (Compound **5**)

White solid; m. p. 142–143 °C. IR(KBr): v_max_ = 2953, 2918, 2848, 1598, 1460, 1378, 1263, 1097, 802 cm^−1^. ^1^H NMR (CDCl_3_, 500 MHz): δ 7.59 (d, J = 9.0 Hz, 6H), 7.37 (s, 3H), 6.99 (d, J = 8.5 Hz, 6H), 3.86 (s, 9H). ^13^C NMR (CDCl_3_, 125 MHz): 159.7, 141.7, 134.0, 129.3, 122.7, 114.2, 55.9.


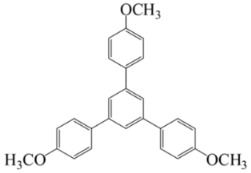


##### 1,3,5-Tris(4-Hydroxyphenyl)Benzene (Compound **6**)

White solid; m. p. 237–239 °C. IR (KBr): v_max_ = 2953, 2918, 2848, 1606, 1460, 1378, 1263, 1097, 802 cm^−1^. ^1^H NMR (CDCl_3_): δ = 3.81 (s, 9H), 6.94–7.41 (m, 12H), 7.79 (s, 3H). ^13^C NMR (CDCl_3_, 125 MHz): δ 55.4, 114.3, 123.8, 128.3, 133.9, 141.8, 159.3.


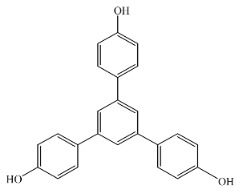


### 4.3. Protein Aggregation

HEWL concentration was determined by optical absorption at 280 nm (ε_280_ = 2.65 L·g^−1^·cm^−1^) [60]. HEWL was dissolved to a concentration of 2 mg/mL (140 µM) in 50 mM glycine buffer, pH 2.5, centrifuged for 10 min at 13,000 rpm, then incubated at 57 °C for the specified time lengths in the absence or presence of the indicated concentrations of compounds **1**–**6**, under gentle stirring at 250 rpm by a Teflon magnetic bar [33].

### 4.4. ThT Fluorescence Assay

A mother solution of 2.5 mM ThT was prepared in 10 mM sodium phosphate buffer, 150 mM NaCl, pH 7.0. It was then passed through a 0.45 μm filter paper and stored at 4 °C until it was placed at room temperature immediately before use. At regular time intervals, a 10 µL aliquot of protein solution was mixed with 990 μL of ThT solution in a 10 × 10 mm quartz cuvette and the fluorescence emission spectra were recorded at room temperature using a Cary Eclipse VARIAN fluorescence spectrophotometer (Varian, Mulgrave, Australia) and an excitation wavelength of 440 nm [61].

### 4.5. CR Assay

Since compounds **1**–**6** have optical absorption spectra partially overlapped to that of CR, all protein samples were centrifuged at 10,000 rpm and the pellets resuspended in 50 mM glycine buffer, pH 2.5, at room temperature. A mother solution of 20 mM CR was prepared in 5 mM sodium phosphate buffer, 150 mM NaCl, pH 7.4, it was then filtered using a center-glass N4 filter and stored at 4 °C, until it was placed at room temperature immediately before use. At different time points during protein aggregation, a 60 μL aliquot of resuspended protein solution was mixed with 440 μL of CR solution. Optical absorption spectra were acquired after 2–3 min equilibration at 25 °C, using a Cecil 7200 UV–visible spectrophotometer (Aquarius, Cambridge, England) and a 1 cm path-length cell.

### 4.6. FTIR Spectroscopy

Samples were incubated for 48 h to promote aggregation as described above in the presence of 0.32 µM compounds **1**–**6**, centrifuged at 13,000× *g* for 10 min and resuspended in D_2_O to a final volume of 72.8 μL, to reach a final concentration of 27.8 mg/mL. For each sample, 40 μL was deposited onto an Omni-Cell transmission cell with a 25 μm spacer (Specac, Orpington, UK), and Fourier-transform infrared (FTIR) spectra were recorded using a FTS-40 FTIR spectrometer (Bio-Rad, Hercules, CA, USA) purged with a continuous flow of dry air. Sixty-four interferograms were accumulated in each case at a spectral resolution of 1 cm^−1^. The FTIR absorbance spectra were blank-subtracted and baseline-corrected to reveal the amide I region (1600–1700 cm^−1^). For each spectrum, the sharp water peaks in the amide I and flanking regions (1580–1800 cm^−1^) were eliminated by using a multiple Gaussian function to fit only the experimental data that were devoid of such peaks in this region and replotting the spectrum as the best fitted function. All spectra were normalized to the same amide I region area. Additional smoothing was not carried out.

### 4.7. Atomic Force Microscopy

HEWL samples pre-incubated for 48 h under aggregating conditions with or without 0.32 µM compounds **1**–**6** were diluted 100 times in filtered deionized water. A 5 µL aliquot of each of the resulting samples was placed on freshly cleaved mica at room temperature. After a few minutes, the mica was slowly washed with 100 μL of deionized water and dried with N_2_. Each image was acquired in a non-contact mode at a scan speed of 30 μm/s, loop filter of 3 Hz and force of 200 nN with a Dual Scope Probe Scanner (Veeco, model Auto probe, CP-Research, CA, USA) with an area of 5 × 5 μm^2^. Conical shape silicon tips (MikroMasch NSC16) with a resonance frequency of 150 kHz and a nominal constant of 40 N/m were used. Fifteen images were collected for each compound.

### 4.8. Cell Cultures

Authenticated human neuroblastoma SH-SY5Y cells were purchased from American Type Culture Collection (ATCC) (Manassas, VA, USA) and they tested negative for mycoplasma contaminations. SH-SY5Y cells were cultured in Dulbecco’s modified Eagle’s medium (DMEM), F-12 Ham with 25 mM HEPES and NaHCO_3_ (1:1) supplemented with 10% fetal bovine serum (FBS), 1.0 mM glutamine and 1.0% penicillin and streptomycin solution. Cells were maintained in a 5.0% CO_2_ humidified atmosphere at 37 °C and grown until 80% confluence for a maximum of 20 passages.

### 4.9. 3-(4,5-Dimethylthiazol)-2,5-Diphenyltetrazolium Bromide (MTT) Reduction Test

HEWL samples pre-incubated for 48 h under aggregating conditions in the absence or presence of 0.32 µM compounds **1**–**6** were centrifuged and the pellets were dried under N_2_ to remove any compounds and DMSO, dissolved in cell culture medium at a final HEWL concentration of 2 μM (monomer equivalents), and added to SH-SY5Y cells seeded in 96-well plates for 24 h at 37 °C. The cell medium was then removed and the MTT solution (0.5 mg/mL in RPMI) was added to the cells for 4 h. The medium was then aspirated, and the formazan product was solubilized with cell lysis buffer (20% SDS, 50% N, N-dimethylformamide, pH 4.7) for 1 h, as previously described [62].

### 4.10. Measurement of Caspase-3 Activity

HEWL aggregates were formed in the absence or in the presence of 0.32 µM of compounds **1**–**6** as described in the previous section and then added to the cell culture medium of SH-SY5Y cells seeded on glass coverslips for 24 h at 2 μM (monomer equivalents). Caspase-3 activity was then analyzed by using FAM FLICA^TM^ caspases 3 & 7 solution (caspase 3 & 7 FLICA kit FAM-DEVDFMK, Immunochemistry Technologies, LLC, Bloomington, MN, USA). Cell fluorescence was analyzed by the TCS SP8 scanning confocal microscopy system (Leica Microsystems, Mannheim, Germany) equipped with an argon laser source, as previously reported [62]. A series of 1.0 μm thick optical sections (1024 × 1024 pixels) was taken through the cell depth for each sample using a Leica Plan Apo 63 × oil immersion objective for fluorescence measurement at 488 nm. The confocal microscope was set at optimal acquisition conditions, e.g., pinhole diameters, detector gain, and laser powers. Settings were maintained constant for each analysis.

### 4.11. Statistical Analysis

All data were expressed as means ± standard error of mean (S.E.M). ANOVA followed by Bonferroni’s post comparison test was used to compare different values.

## 5. Conclusions

In conclusion, we have provided experimental evidence of different effects of various substituents of the architecture of the 1,3,5-triarylbenzene molecule on the inhibition of the self-assembly of HEWL. We showed that –OH, –OCH_3_, and –CH_3_ groups as electron-donor groups positioned on aromatic rings have a low efficacy, whereas –F and –NO_2_ as electron-withdrawing groups have great efficacy in inhibiting amyloid formation by HEWL. Based on the results of this study and others, it is apparent that small aromatic compounds and functional groups appended on these aromatic molecules can have a profound effect on their ability to inhibit amyloid formation. These results may ultimately find applications in the development of potential amyloid inhibitors and, thus, possible use of these compounds as a therapeutic approach in the treatment of amyloid deposition diseases.

## Supplementary Materials

Supplementary materials can be found at https://www.mdpi.com/1422-0067/20/22/5558/s1.

## Abbreviations

HEWL	hen egg white lysozyme
ThT	thioflavin T
HPA/NCP	H_6_P_2_W_18_O_62_/nanoclinoptilolite
CR	Congo red
AFM	atomic force microscopy
FTIR	Fourier-transform infrared spectroscopy
MTT	3-(4,5-dimethylthiazol)-2,5-diphenyltetrazolium bromide
NMR	nuclear magnetic resonance
TLC	thin layer chromatography
DMSO	dimethyl sulfoxide
mp	melting point
DMEM	Dulbecco’s modified Eagle’s medium
PBS	phosphate buffered saline
