# Supplementary material for: Identification of Novel 1,3,5-Triphenylbenzene Derivative Compounds as Inhibitors of Hen Lysozyme Amyloid Fibril Formation
Source: Int J Mol Sci. 2019 Nov 7;20(22):5558. doi: 10.3390/ijms20225558 (PMC6888386; doi:10.3390/ijms20225558)
Supplement: Supplementary file 1 [file ijms-20-05558-s001.pdf]

Electronic Supporting Information

Compound 1: 1,3,5-Triphenylbenzene

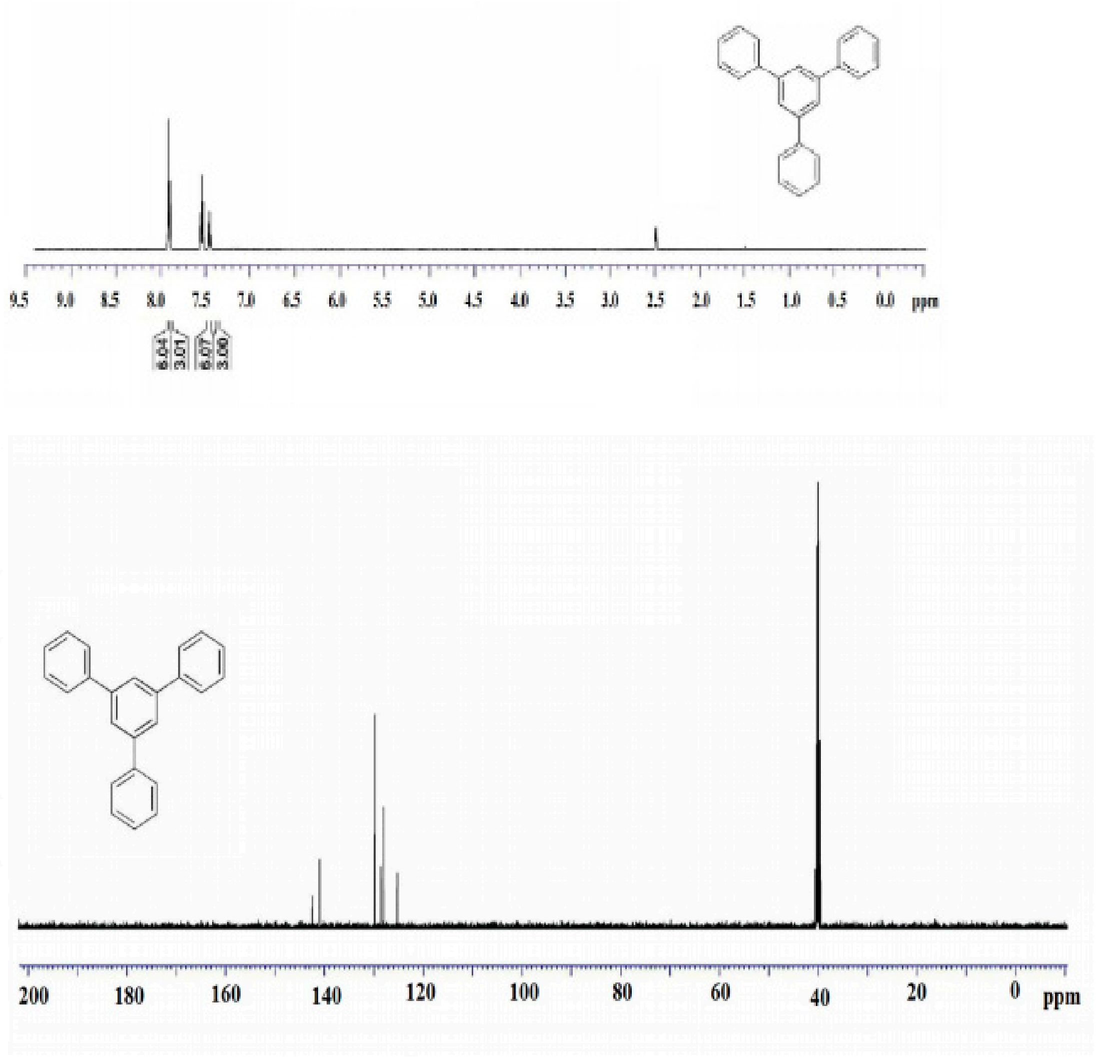

Figure S1. ^{1}H NMR spectrum (top) and ^{13}C NMR spectrum (bottom) of **Compound 1.**

Compound 2: 1,3,5-Tris(4-fluorophenyl)benzene

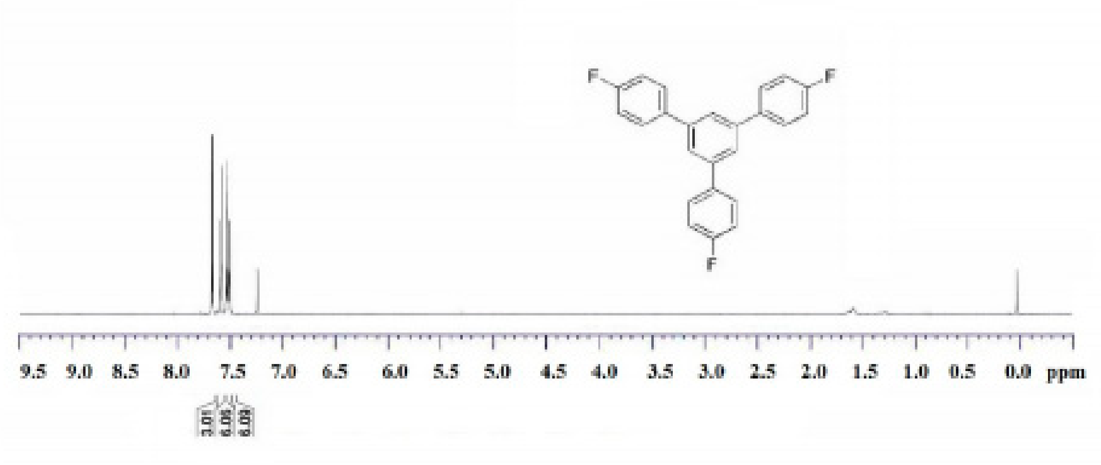

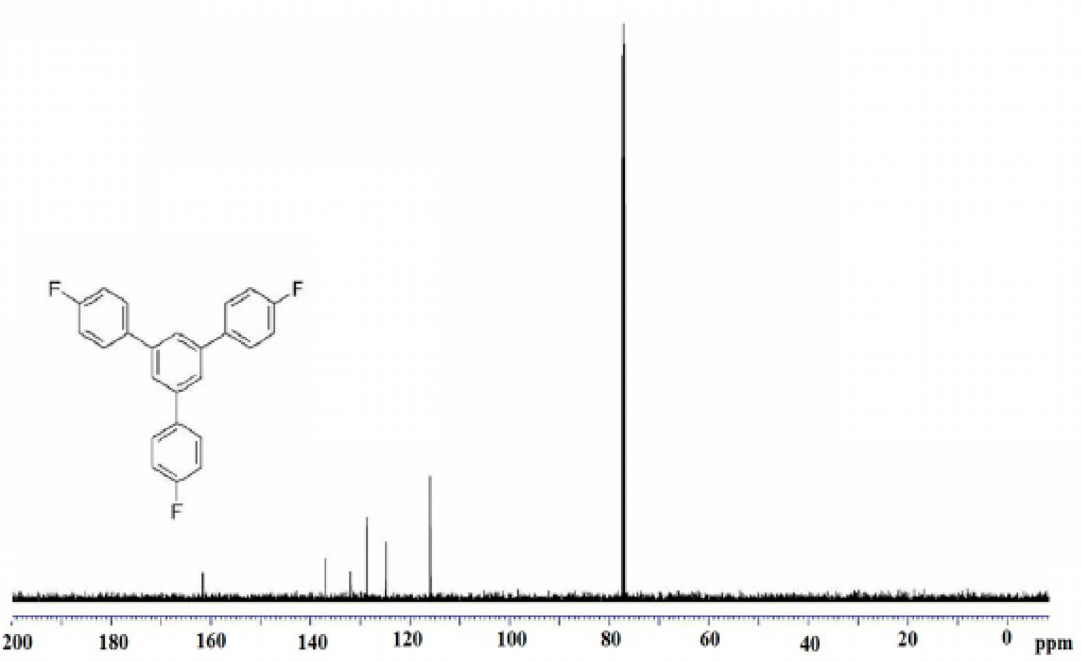

Figure S2. ^{1}H NMR spectrum (top) and ^{13}C NMR spectrum (bottom) of **Compound 2.**

Compound 3: 1,3,5-Tris(4-nitrophenyl)benzene

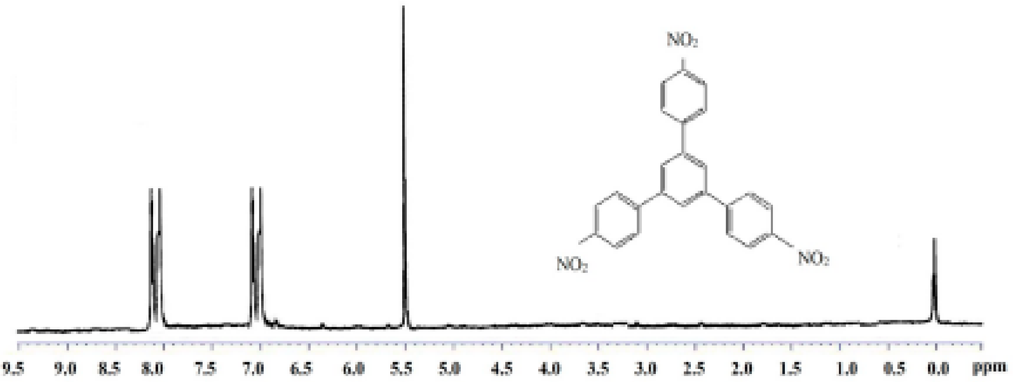

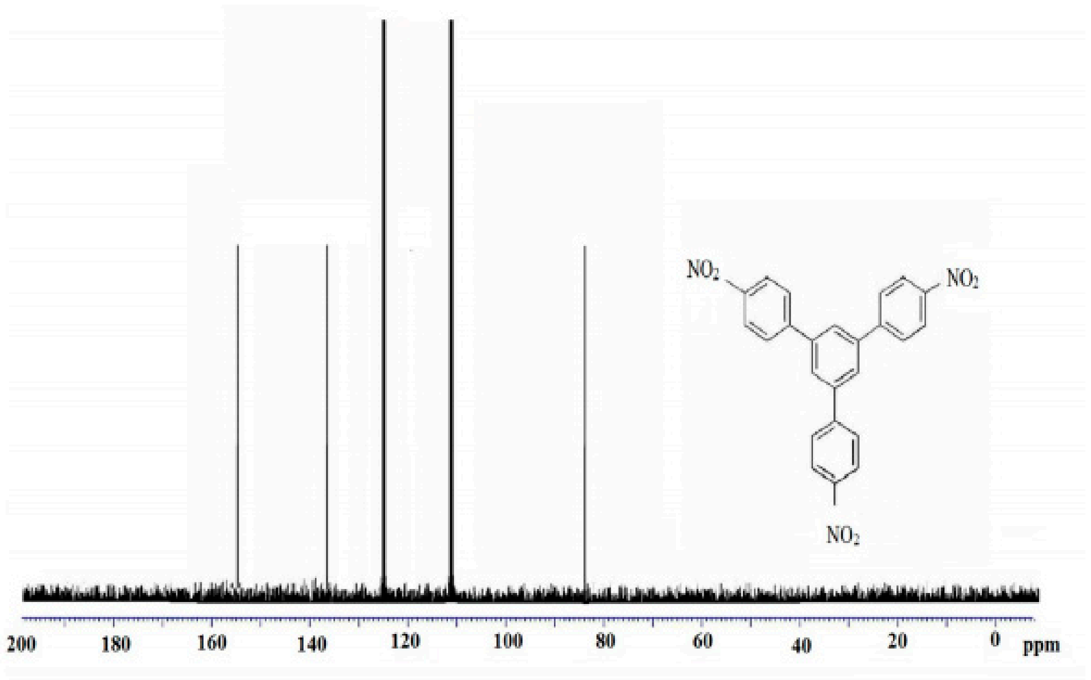

Figure S3. ^{1}H NMR spectrum (top) and ^{13}C NMR spectrum (bottom) of **Compound 3.**

Compound 4: 1,3,5-Tris(4-methylphenyl)benzene

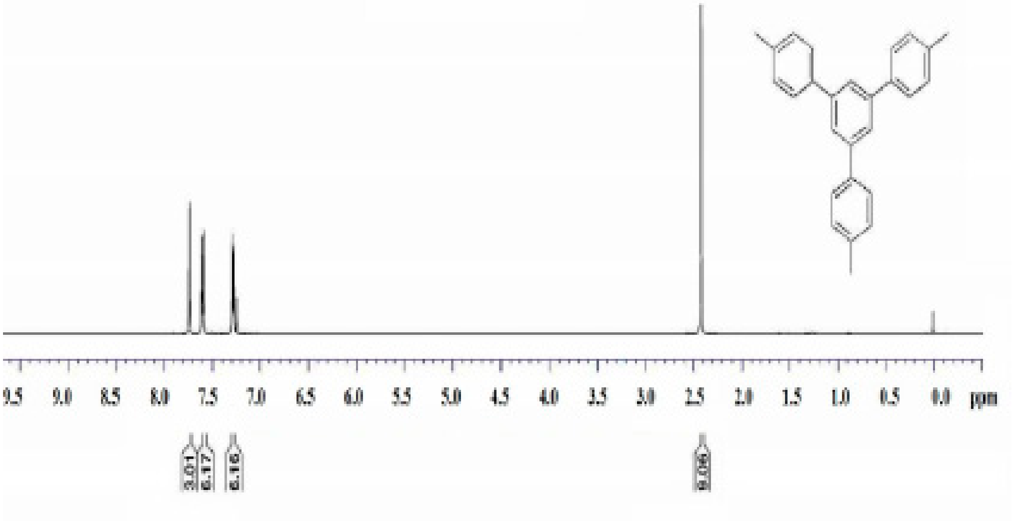

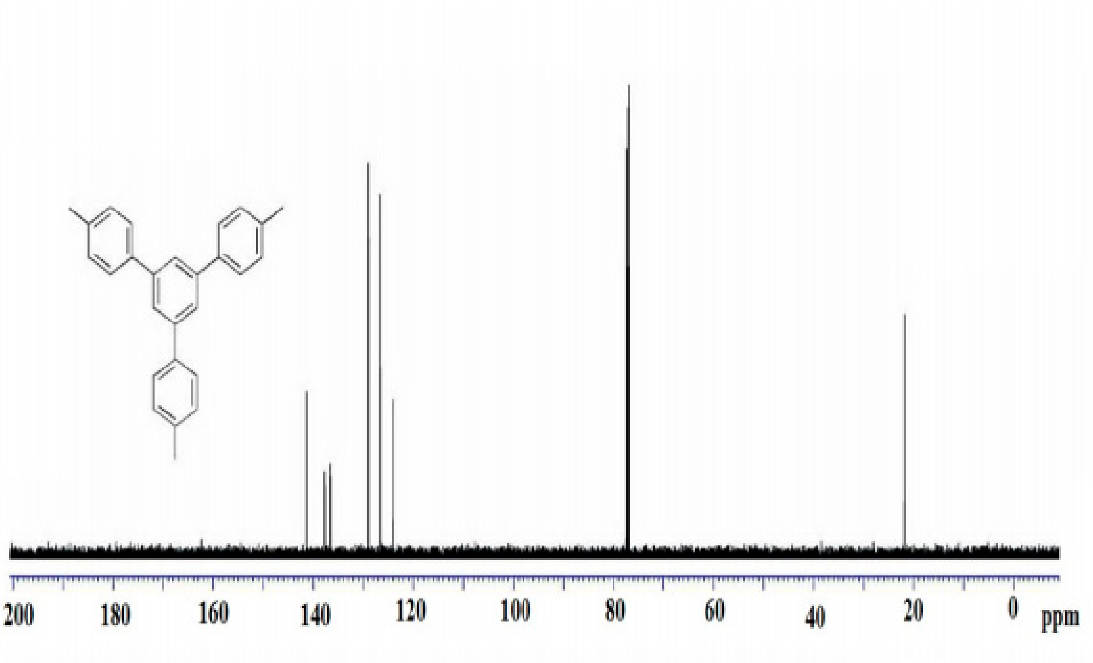

Figure S4. ^{1}H NMR spectrum (top) and ^{13}C NMR spectrum (bottom) of **Compound 4.**

Compound 5: 1,3,5-Tris(4-methoxyphenyl)benzene

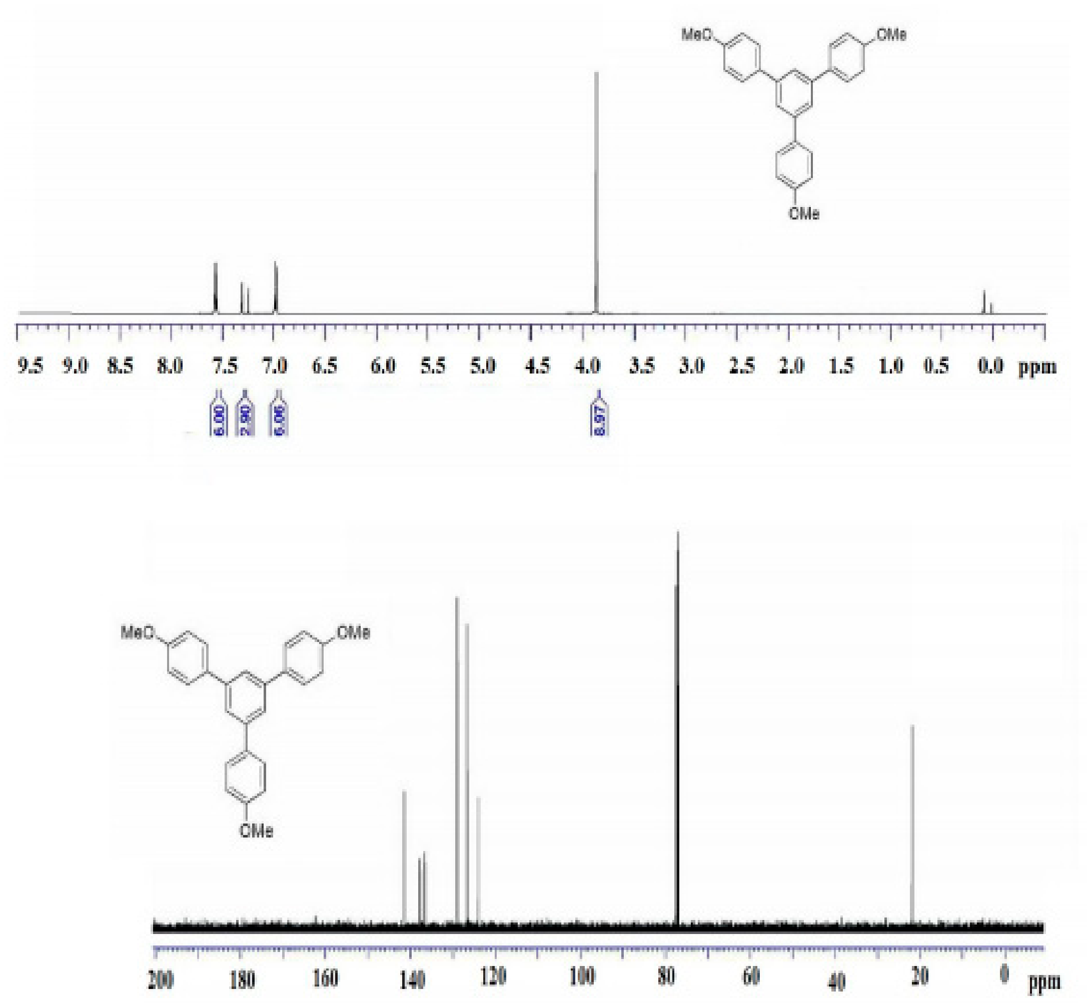

Figure S5. ^{1}H NMR spectrum (top) and ^{13}C NMR spectrum (bottom) of **Compound 5.**

Compound 6: 1,3,5-Tris(4-hydroxyphenyl)benzene

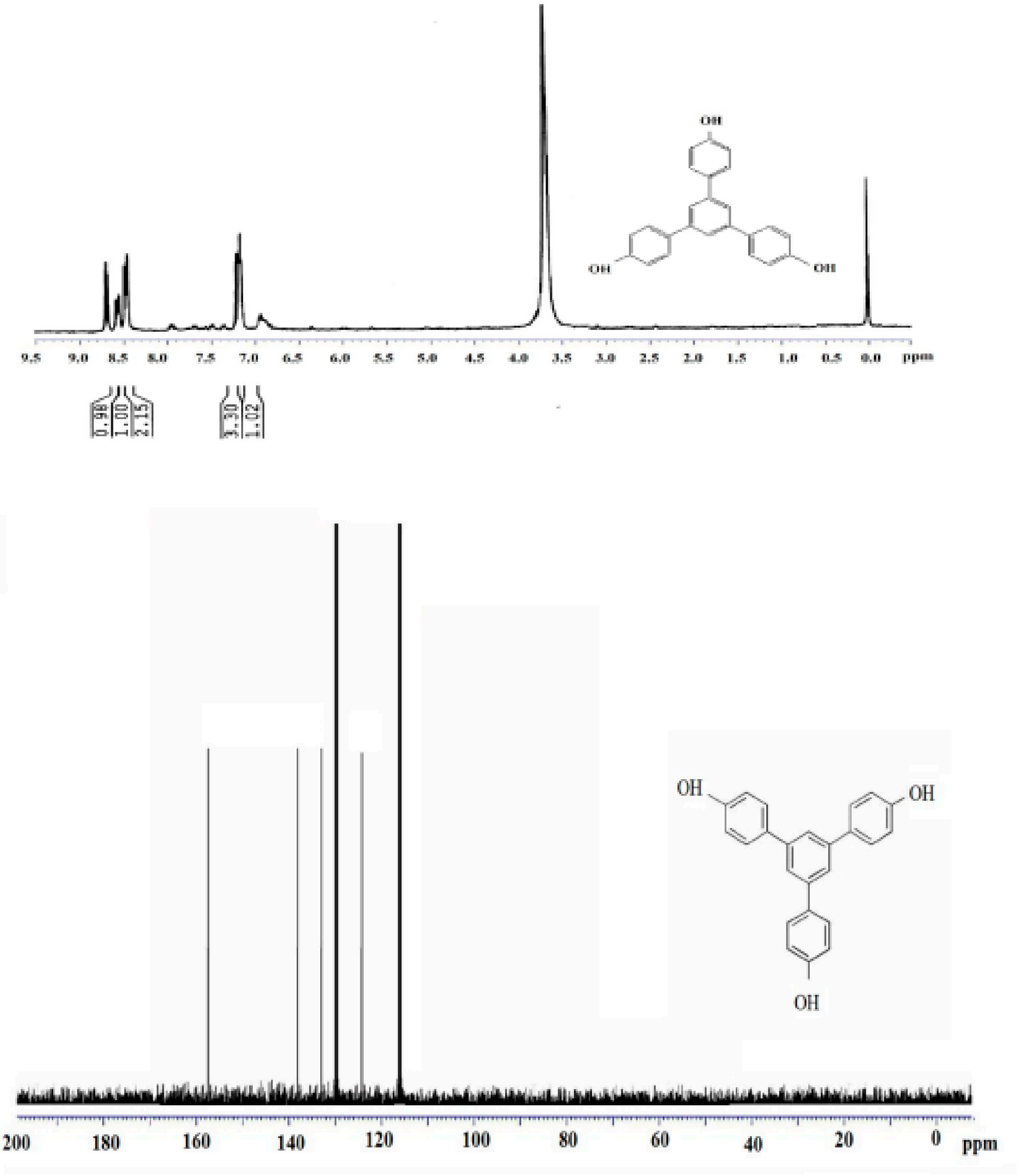

Figure S6. ^{1}H NMR spectrum (top) and ^{13}C NMR spectrum (bottom) of **Compound 6.**

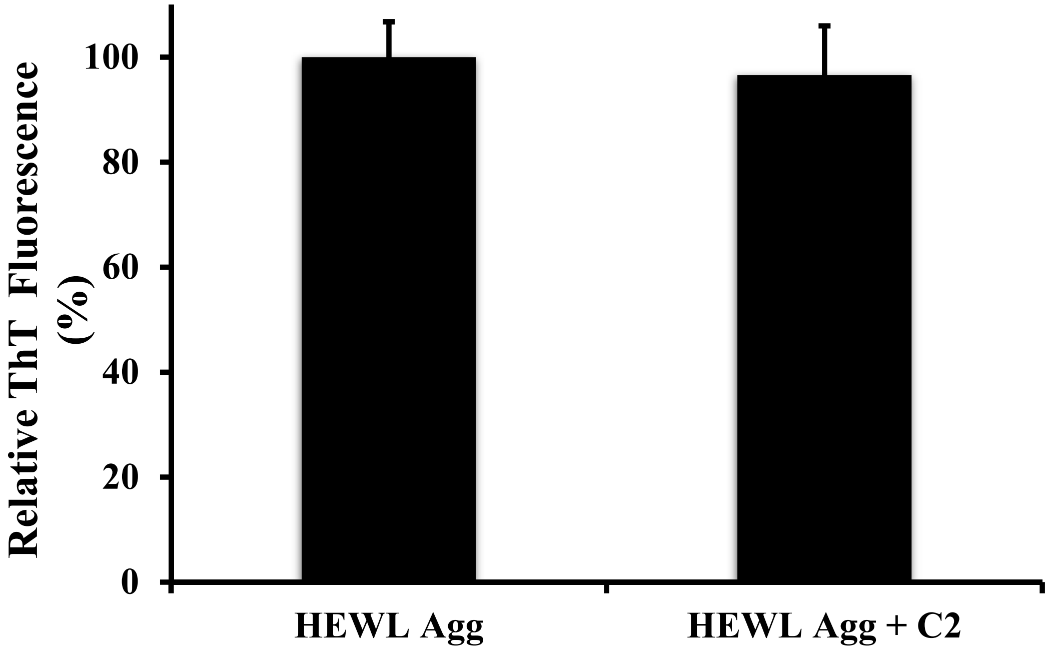

Figure S7. Study of the quenching activity of ThT fluorescence by compound 2. HEWL aggregates were obtained after 48 h incubation at pH 2.5 and 57 °C under stirring (250 rpm) and immediately monitored by the ThT fluorescence assay before and after adding compound 2 at a final concentration of 0.32 μm.

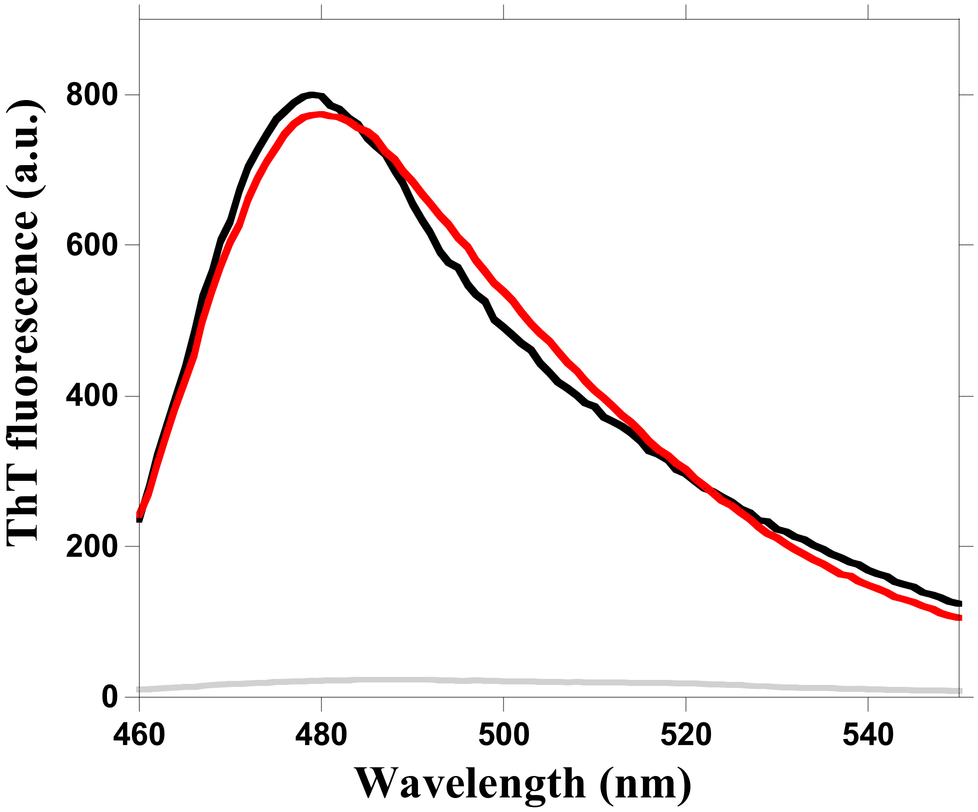

Figure S8. Effect of DMSO, at a concentration of 2% (*v*/*v*), on HEWL aggregation at 2 mg/ml, in 50 mM glycine buffer, pH 2.5, 57 °C, under stirring (250 rpm), after 48 h incubation monitored by changes in ThT florescence intensity. The spectra refer to ThT alone (gray), HEWL pre-incubated with 0 % (black) and 2% (*v*/*v*) DMSO (red).
